# Oxidative DNA damage triggers ProNGF-Mediated apoptosis in the striatum of 6-OHDA-treated rats

**DOI:** 10.1186/1750-1326-7-S1-S20

**Published:** 2012-02-07

**Authors:** Tao Wang, Pingping Zuo, Piu Chan

**Affiliations:** 1 Department of Neurobiology, Beijing Institute of Geriatrics, Xuanwu Hospital of Capital Medical University, Beijing 100053, China; 2 Department of Pharmacology, Institute of Basic Medical Sciences, Peking Union Medical College & Chinese Academy of Medical Sciences, Beijing, China

## Background

Oxidative DNA damage generated by 6-hydroxydopamine (6-OHDA) may initiate a programmed cell death cascade called apoptosis and plays an important role in the pathogenesis of Parkinson’s disease (PD). ProNGF, a precursor of nerve growth factor (NGF), is found to be involved in neuronal apoptosis of neurodegenerative diseases such as PD. Therefore, we have tested the presumption that proNGF-mediated apoptosis might be initiated by oxidative DNA damage in 6-OHDA treated rats.

## Method

Enzymatic assay, histochemistry, TUNEL and Western blot were used to investigate profiles of pro-NGF mediated apoptosis after oxidative DNA damage in a unilateral 6-OHDA treated rat model of PD.

## Result

A signaling cascade of proNGF-mediated apoptosis definitely occurred in striatum of 6-OHDA lesioned rat under a circumstance of oxidative DNA damage. We confirmed the following changes: 1) activities of antioxidant defense system and levels of malondialdehyde (MDA), 2) MTH1 levels and 8-oxo-2’-deoxyguanosine (8-oxo-dG) accumulation, 3) loss of the TH-positive fiber and dopamine neurons in nigrosriatal system, 4) appearance of apoptotic body and neuronal apoptosis in striatum, 5) expression of proNGF and downstream co-receptor of sportily and p75NTR, 6) activation status of c-Jun N-terminal kinase (JNK) and its target p53, 7) activation status of the intrinsic apoptotic pathway including cytochrome *c*, caspase 9 and caspase 3, 8) phosphorylation levels of Bad and ratio of Bax/Bcl-2.

## Conclusion

These results support that after exposure to the stress stimuli such as oxidative DNA damage induced by 6-OHDA, the destiny of neuronal cells in striatum finally went to apoptosis mediated by proNGF-mediated signaling and it suggests that these profiles might provide some clues for the possible molecular basis of PD etiology.

